# Naringenin-Functionalized Multi-Walled Carbon Nanotubes: A Potential Approach for Site-Specific Remote-Controlled Anticancer Delivery for the Treatment of Lung Cancer Cells

**DOI:** 10.3390/ijms21124557

**Published:** 2020-06-26

**Authors:** Renata P. Morais, Gabrielle B. Novais, Leandro S. Sangenito, André L. S. Santos, Ronny Priefer, Margreet Morsink, Marcelo C. Mendonça, Eliana B. Souto, Patrícia Severino, Juliana C. Cardoso

**Affiliations:** 1 Tiradentes University (UNIT), 300, Murilo Dantas Ave, Farolândia 49032-490, Brazil; Renatamorais04@hotmail.com (R.P.M.); gabibarrozonovais@gmail.com (G.B.N.); marcelodacostamendonca@gmail.com (M.C.M.); 2 Technology and Research Institute (ITP), 300, Murilo Dantas Ave, Farolândia 49032-490, Brazil; 3 Federal University of Rio de Janeiro (UFRJ), 373, Carlos Chagas Filho Ave, Cidade Universitária da Universidade Federal do Rio de Janeiro, Rio de Janeiro 21941-901, Brazil; ibastefano@hotmail.com (L.S.S.); andre@micro.ufrj.br (A.L.S.S.); 4 School of Pharmacy, Massachusetts College of Pharmacy and Health Sciences, 179 Longwood Avenue, Boston, MA 02115, USA; ronny.priefer@mcphs.edu; 5 Center for Biomedical Engineering, Department of Medicine, Brigham and Women’s Hospital, Harvard Medical School, 65 Landsdowne Street, Cambridge, MA 02139, USA; m.a.j.morsink@student.utwente.nl; 6 Translational Liver Research, Department of Medical Cell BioPhysics, Technical Medical Centre, Faculty of Science and Technology, University of Twente, 7522 NB Enschede, The Netherlands; 7 Department of Developmental BioEngineering, Faculty of Science and Technology, Technical Medical Centre, University of Twente, 7522 NB Enschede, The Netherlands; 8 Department of Pharmaceutical Technology, Faculty of Pharmacy, University of Coimbra, Pólo das Ciências da Saúde, Azinhaga de Santa Comba, 3000-548 Coimbra, Portugal; 9 CEB-Centre of Biological Engineering, University of Minho, Campus de Gualtar, 4710-057 Braga, Portugal; 10 Tiradentes Institute, 150 Mt Vernon St, Dorchester, MA 02125, USA

**Keywords:** carbon nanotubes, naringenin, flavanones, functionalization, antineoplastic agents, lung cancer

## Abstract

Multi-walled carbon nanotubes functionalized with naringenin have been developed as new drug carriers to improve the performance of lung cancer treatment. The nanocarrier was characterized by Transmission Electron Microscopy (TEM), Fourier-Transform Infrared Spectroscopy (FTIR), X-ray photoelectron spectroscopy, Raman Spectroscopy, and Differential Scanning Calorimetry (DSC). Drug release rates were determined in vitro by the dialysis method. The cytotoxic profile was evaluated using the MTT assay, against a human skin *cell line* (*hFB*) as a model for normal cells, and against an adenocarcinomic human alveolar basal epithelial (A569) cell line as a lung cancer in vitro model. The results demonstrated that the functionalization of carbon nanotubes with naringenin occurred by non-covalent interactions. The release profiles demonstrated a pH-responsive behavior, showing a prolonged release in the tumor pH environment. The naringenin-functionalized carbon nanotubes showed lower cytotoxicity on non-malignant cells (hFB) than free naringenin, with an improved anticancer effect on malignant lung cells (A549) as an in vitro model of lung cancer.

## 1. Introduction

Lung cancer is one of the most common malignant tumors worldwide [1], occurring either as small-cell lung carcinoma (SCLC) or non-small cell lung carcinoma (NSCLC) [2]. Clinically, both neoplasms are characterized as asymptomatic in the initial stage, which leads to late diagnosis when the disease is in a locally advanced or metastatic stage, resulting in high mortality rates [3,4].

Surgery plays an important role when a tumor is detected at an early stage, increasing the survival of patients. Unfortunately, when the disease is already in an advanced stage, surgery may no longer be an option. Chemotherapy is therefore the primary treatment to relieve symptoms and prolong the lifetime of a patient. However, the physiological and pathological characteristics of the lungs, the high variability of the tumor microenvironment, and the malignant and resistant effects on cancer cells after chemotherapy, are some limitations encountered in conventional chemotherapy of lung carcinomas [4,5].

Nanotechnology has the potential to overcome these limitations. Amongst a range of nanomaterials, carbon nanotubes (CNTs) have attracted much attention due to their ability to interact with therapeutic agents, making them an excellent candidate in the development of therapies employing specific targeting for the treatment of tumors [6]. CNTs are tailored as carbon atoms arranged in condensed aromatic rings formed by layers of graphene rolled into a cylindrical shape [7,8]. CNTs exhibit a high surface area, very low density, high aspect ratio, favorable electrical properties, and chemical stability. However, CNTs show high hydrophobicity and risk of agglomeration, due to the van der Waals interactions [9]. Moreover, the risk of toxicity of CNTs has been attributed to their shape and patterns [3]. To decrease their hydrophobicity and toxicological risk, surface modification by the attachment of specific functional groups or molecules through their walls or extremities has been proposed [10,11]. The high surface area of CNT allows the functionalization of a large number of molecules and facilitates the pathway through biological membranes by passive diffusion and/or endocytosis, favoring the accumulation in intracellular compartments [12,13].

Naringenin (Ngn) is a flavanone obtained from citrus fruits, which exhibits a wide range of pharmacological effects, including potent anti-cancer [14,15], antioxidant, anti-inflammatory, and hepatoprotective activity [16,17]. Ngn has been reported to induce apoptosis and decreases the viability of several cancer cell lines, including lung cancer [18]. The anti-cancer activity occurs due to the capacity of signaling in cell proliferation, the arrest of the cell cycle and induction of apoptosis, as well as DNA repair mechanisms within carcinogenic cells [16,19]. A variety of in vitro studies reports the consistent antiproliferative and/or anti-migratory effects of Ngn in a variety of cancer and tumor cell lines.

In this work, we propose the surface functionalization of CNT with Ngn using a process in a non-oxidative environment, for site-specific delivery to tumor lung cells. We intend to obtain a simple process that produces a nanocomposite formulation able to improve the drug delivery in the tumor site, presenting biocompatibility on non-tumor cells and tumor cell decrease in proliferation. The developed Ngn-functionalized CNT represent a technological innovation suitable for antitumor therapeutic strategies.

## 2. Results

**Figure 1:**
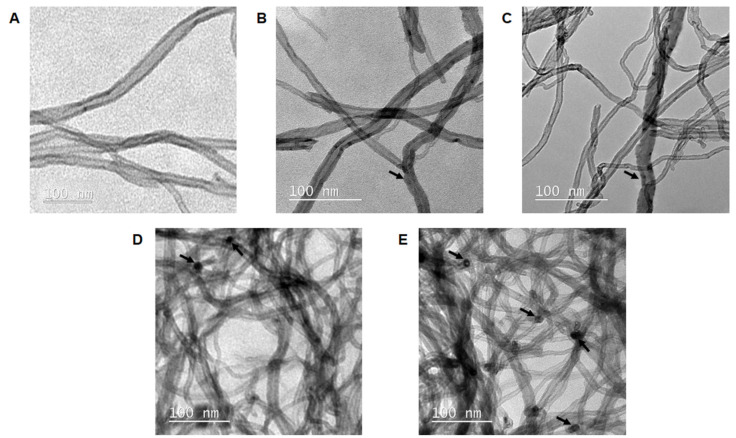
TEM images of (**A**) CNT (carbon nanotube), (**B**) CNA (CNT after acid treatment), (**C**) CNA(ox) (CNA submitted to oxidative environment), (**D**) CNANgn(ox) (CNA treated with naringenin in oxidative environment) and (**E**) CNANgn (CNA treated with naringenin).

Figure 1 shows the TEM images of the samples. The calculated medium diameter of CNT, CNA (CNT after acid treatment), CNA(ox) (CNA submitted to oxidative environment), CNANgn(ox) (CNA treated with naringenin in oxidative environment), and CNANgn (CNA treated with naringenin) were 7.1 ± 2.3 nm, 8.8 ± 2.0 nm, 7.7 ± 1.7 nm, 9.4 ± 2.3 nm, and 9.6 ± 1.4 nm, respectively. CNT showed a smooth surface with few defects on the side walls (Figure 1A). After acid treatment, CNA showed defects in the side walls, such as roughness (arrows). However, the tubular morphology was maintained even after oxidation (Figure 1B). The morphological characteristics of the CNA(ox) were similar to CNA (Figure 1C). CNANgn and CNANgn(ox) had dark particle-like spots located along the side walls of the samples (arrows) (CNANgn(ox), Figure 1D; CNANgn, Figure 1E).

**Figure 2:**
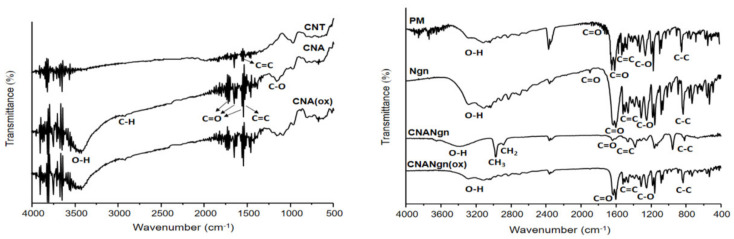
FTIR (Fourier-Transform Infrared Spectroscopy) spectra of CNT (carbon nanotube), CNA (CNT after acid treatment), CNA(ox) (CNA submitted to oxidative environment), CNANgn (CNA treated with naringenin), CNANgn(ox) (CNA treated with naringenin in oxidative environment), PM (physical mixture of CNA and naringenin) and Ngn (naringenin).

The Fourier-Transform Infrared Spectroscopy (FTIR) spectrum (Figure 2) of the CNT showed a band at 1550 cm^−1^. The CNA spectrum showed bands at 3400 cm^−1^, 2949 cm^−1^, between 1711 and 1550 cm^−1^, and between 1050 and 1300 cm^−1^. In the CNA(ox) spectrum, the same bands were observed; however, their intensities were slightly decreased. Ngn’s FTIR matched that as reported in the literature [20]. The CNANgn spectrum presented a displacement of the band in 3293 to 3396 cm^−1^ and 1630 to 1639 cm^−1^, with an increase in energy absorption. In addition, disappearance of the band at 1898 cm^−1^ and presence of the 2980 cm^−1^ and 2884 cm^−1^ bands were observed. The CNANgn(ox) spectrum showed all bands in approximately the same positions as the Ngn bands.

**Figure 3:**
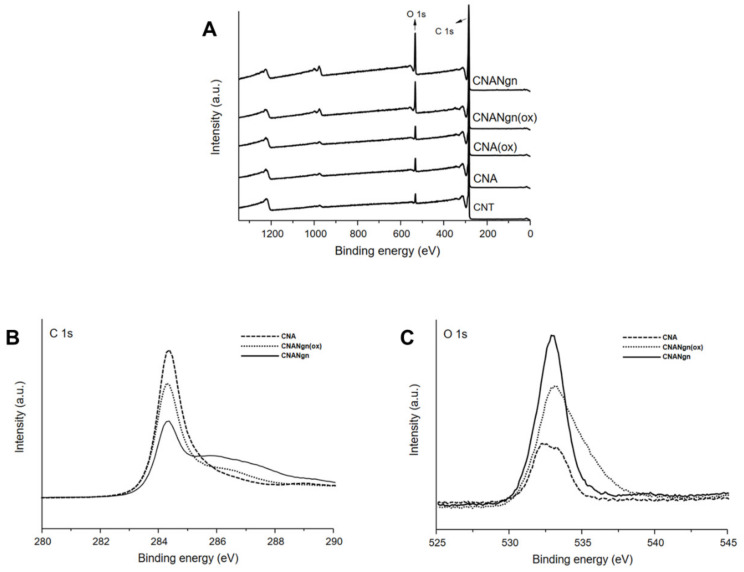
(**A**) XPS survey spectra of CNT (carbon nanotube), CNA (CNT after acid treatment), CNA(ox) (CNA submitted to oxidative environment), CNANgn (CNA treated with naringenin) and CNANgn(ox) (CNA treated with naringenin in oxidative environment). (**B**) The binding energy of C1s spectra. (**C**) The binding energy of O1s spectra.

XPS was performed on all CNT samples. Samples showed the carbon signal (C1*s*) and the oxygen signal (O1*s*) at 284 and 533 eV, respectively. The intensity of the oxygen signal (O1*s*) increased according to the sequence: CNT, CNA, CNA(ox), CNANgn(ox), and CNANgn (Figure 3A). CNANgn showed a displacement of the peak C1*s* and O1*s* to higher and lower binding energy, respectively (Figure 3B,C). The first peak corresponds to the C-C, while C-H components of the hexagonal structure of the carbon nanotube and the aromatic structure of the Ngn. The second one appeared as a broad peak and is attributed to the groups C-O and C=O of the structure of the Ngn (Figure 3B) [21]. CNANgn(ox), despite showing an increase in oxygen signal, did not present displacement of the peak C1s and O1s.

**Figure 4:**
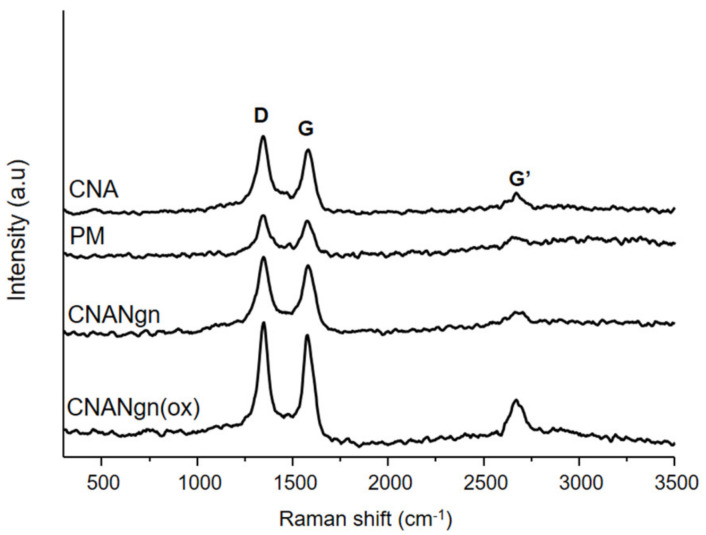
Raman spectra of CNA (CNT after acid treatment), CNANgn (CNA treated with naringenin), CNANgn(ox) (CNA treated with naringenin in oxidative environment) and PM (physical mixture of CNA and naringenin).

The Raman spectra of the CNA showed typical signals at 1346 and 1568 cm^−1^, corresponding to bands D and G, respectively (Figure 4). The D and G bands were of different intensities and I_D_/I_G_ ratios. The I_D_/I_G_ ratios for CNA, physical mixture (PM), CNANgn, and CNANgn(ox) were 1.16, 1.1, 1.09, and 1.02, respectively. The peak frequency of the G band of CNANgn (1576 cm^−1^) and CNANgn(ox) (1572 cm^−1^) were slightly higher than the frequency for CNA (1568 cm^−1^) and PM (1568 cm^−1^).

**Figure 5:**
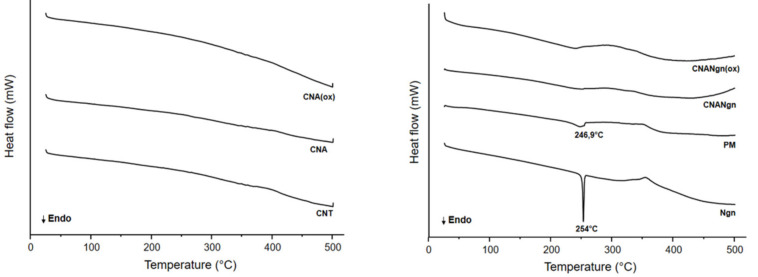
Differential scanning calorimetry (DSC) of CNT (carbon nanotube), CNA (CNT after acid treatment), CNA(ox) (CNA submitted to oxidative environment), CNANgn (CNA treated with naringenin), CNANgn(ox) (CNA treated with naringenin in oxidative environment), PM (physical mixture of CNA and naringenin) and Ngn (naringenin).

The DSC profile of CNT, CNA, and CNA(ox) samples did not show any enthalpic event up to 500 °C. Ngn exhibited an endothermic peak at starting at 251.7 °C and completing at 255.2 °C (Table 1), followed by an exothermic peak (~360 °C). However, these peaks were less perceived in the CNANgn and CNANgn(ox) curves. The PM curve also showed these high peaks, however, at slightly lower temperatures (246.9 and 350 °C, respectively) (Figure 5).

**Figure 6:**
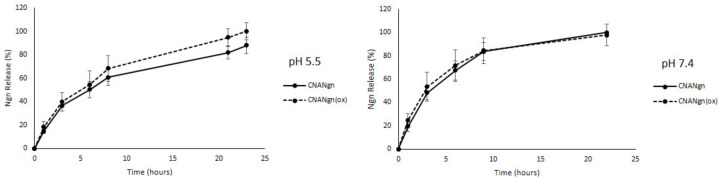
Ngn (naringenin) concentration released (µg/mL) from CNANgn (CNA treated with Ngn) and CNANgn(ox) (CNA treated with Ngn in oxidative environment) at pH 5.5 and 7.4. The data represents the mean ± standard deviation of *n* = 3.

The release of Ngn from the samples (CNANgn and CNANgn(ox)) was faster in the first 6 h in both pH 5.5 and 7.4 media, exhibiting a similar release profile for both samples. At 6 h and 9 h, in the pH 7.4 for CNANgn, the total released of Ngn was 60.8% and 83.5%, while at pH 5.5 it was 50.4% and 60.8, respectively (*p* < 0.05). For CNANgn(ox) at 6 h and pH 7.4, total released Ngn was 71.8% compared to 54.4% at pH 5.5 (*p* < 0.05). All values had no significant differences (*p* > 0.05). Subsequently, at both pH solutions and CNANgn’s, the release of Ngn slowed until 24 h. In general, at pH 7.4, the release rate was faster than at pH 5.5 (Figure 6). For both samples, regardless of the pH conditions, the Korsmeyer–Peppas model described the system best (r^2^ = 1), showing the *n* value of 1 (Table 2).

**Figure 7:**
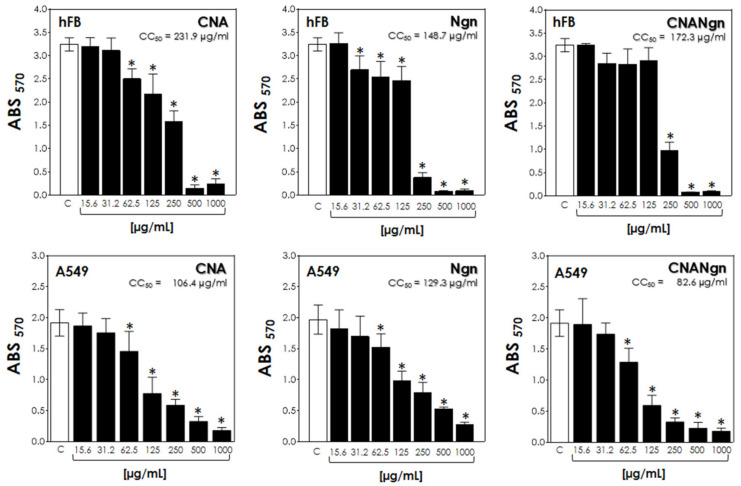
Cell viability of hFB (**top**) cells and A549 (**bottom**) cells treated with CNA (CNT after acid treatment), Ngn (naringenin) and CNANgn (CNA treated with naringenin) tested by MTT assay at a series of concentrations after 48 h. The data represents the mean ± standard deviation of *n* = 3; * significant difference comparing the different concentrations (*p* < 0.05).

CNA and Ngn did not affect the viability of hFB cells (non-malignant cells) at low concentrations (up to 15.6 µg/mL and 31.2 µg/mL, respectively), as seen in Figure 7. CNANgn did not show significant cytotoxicity until a concentration of 125 µg/mL was reached. The CC_50_ for CNA, Ngn, and CNANgn were 231.9 ± 17.4 µg/mL, 148.7 ± 14.1 µg/mL, and 172.3 ± 12.9 µg/mL, respectively. Otherwise, the samples CNA, Ngn, and CNANgn showed significant cytotoxicity against human lung alveolar adenocarcinoma cell line (A549) at a concentration of 62.5 µg/mL. Additionally, the CC_50_ of CNANgn was lower (82.6 ± 7.3 µg/mL) than the CC_50_ of CNA (106.4 ± 10.9 µg/mL) and Ngn (129.3 ± 6.0 µg/mL) (Figure 7).

## 3. Discussion

The oxidation step in the CNT functionalization process occurs by acid treatment. Oxidation alters the surface of the CNT and allows the incorporation of functional groups (-COOH and -OH) [1]. However, this treatment is not enough to guarantee the dispersibility and biocompatibility of CNT. Most studies about the functionalization process use polymers, which are used as spacers, improving the dispersibility and biocompatibility of CNT [2]. In this approach, the drugs are anchored to the reactive polymeric sites, requiring one more step for production of the drug delivery system.

New proposals are developed in order to obtain an efficient and simplified strategy for CNT functionalization, such as the use of the drug as a functionalizing agent, which can achieve adequate stability and, at the same time, obtain a drug delivery system.

In this work, we propose the treatment of the CNT with Ngn using a functionalization process in two environments (oxidative and non-oxidative), to obtain a drug delivery system to the lung tumoral cells. The redox reagents decrease the pH of the reaction medium, while the non-oxidative environment maintains a neutral pH. In the acid medium, the protonation state of CNT and Ngn are changed, non-favoring the interaction between them. This event was investigated in order to obtain a simple process that produced a nanocomposite that presents biocompatibility on non-tumor cells and decreases the proliferation of tumor cells.

The morphological structure of the samples and the surface modification of the CNT after functionalization were shown by TEM. The functionalization of the CNT using Ngn promoted the increase in the diameter of the samples, confirming the successful functionalization. The increased diameter can be attributed to the fixation of organic portions on the surface of the CNT [22]. A significant difference in the morphology of CNA and CNA(ox) compared to CNT was also observed. These surface modifications translate the functionalization step generating both chemical and morphological changes [23]. Chemical oxidation caused defects on the sidewalls and at the ends of the nanotubes [23,24]. The increase in CNA deformation was attributed to the adhesion of the functional groups [25], for the Ngn binding. The high surface area of the CNT allows the attachment of a large number of molecules, making it a potential drug carrier [12,13]. The dark spots found in CNANgn and CNANgn(ox) (Figure 1) suggest the presence of Ngn on the surface of the CNA.

FTIR studies also confirmed the functionalization of the CNT. The sidewalls of the CNT present carbon with sp^2^ hybridization in a hexagonal arrangement [26]. In the CNT spectrum, a band at 1550 cm^−1^ was observed corresponding to the vibrations of the C=C elongation, showing an intact structure. The presence of the band at 3400 cm^−1^ in the CNA and CNA(ox) spectra was attributed to the different hydroxyl groups resulting from the acid treatment step. The bands observed between 1711 and 1550 cm^−1^ in the spectra were attributed to the C=O of carboxylic acids and ketones/quinones. The bands between 1050 and 1300 cm^−1^ correspond to the vibrations of C-O and O-H, likely from alcohols, ether, and oxidative anhydrides or carboxylic products from the outer layers of the CNA. The band at 2949 cm^−1^ corresponds to the symmetrical elongation of the C-H connections. The functionalization eliminated the symmetry of the CNT, increasing the generation of induced electrical dipoles and detected signals [27]. Ngn’s FTIR matched that as reported in the literature [20]. The Ngn spectrum showed characteristic absorption bands, which can be attributed to the skeletal vibration of the aromatic compound, which belongs to the flavanone family [28]. The Ngn structure contains three hydroxyls, one carbonyl, and one ethereal functional group, each with the potential to form intermolecular hydrogen bonds [29]. In the CNANgn spectrum, we observed the displacement of the band of the free hydroxyl group (OH) from 3293 to 3396 cm^−1^ and of the carbonyl band (C=O) from 1630 to 1639 cm^−1^. Additionally, there was no band in 1898 cm^−1^ (C=O), suggesting that there was a physical–chemical interaction between CNA and the drug. The possible explanation is the formation of hydrogen bonds between the OH group from Ngn and the C=O from CNA, or vice versa. According to the literature, the displacement of the band related to the stretching vibration of the hydroxyl group (O-H) and carbonyl (C=O) to higher frequencies is due to the intermolecular hydrogen bond [30,31,32]. A band at 1470 cm^−1^ confirmed the presence of Ngn in the sample, which was attributed to aromatic C=C elongation vibrations from the flavonoid aromatic rings [21]. The presence of the 2980 cm^−1^ and 2884 cm^−1^ bands is related to the CH_3_ and CH_2_ stretching vibrations, respectively. It is generally assumed that these groups are located in defective locations on the sidewall surface of CNT [33,34]. Nonetheless, the spectrum of CNANgn(ox) showed the same Ngn bands, with no noticeable changes in the spectrum, suggesting an interaction between Ngn and CNA.

XPS was used to assess the interactions between CNA and Ngn based on the chemical and electronic states on the surface structure of CNT. The increase in the carbon signal (C1*s*) and the oxygen signal (O1*s*) in all samples demonstrated that the acid treatment was able to modify the surface of the CNT, generating oxygenated groups (carboxyl, hydroxyls, ketones, and quinones) on its surface. In addition, the increase in the intensity of the signals suggested the functionalization of the surface of the CNA by the Ngn, reaffirming the results obtained by FTIR and TEM. According to Wang et al., [33] it is possible to predict the type of interaction between the CNT and a molecule by displacing the peaks in the XPS spectrum of the chemical elements involved in the interaction to a higher or lower binding energy [35]. The displacement of the C1*s* peak in CNANgn to a higher binding energy suggested that Ngn was adsorbed to the surface of the CNA by π–π stacking. However, the displacement of the O1*s* peak in CNANgn to a lower binding energy suggested the formation of a hydrogen bond between the Ngn and the oxygenated groups on the surface of the CNA. CNANgn(ox) presented intermediate behavior, pointing to lower interaction when compared to CNANgn. The presence of oxidative and acid environments changes the protonation state of both molecules and can make this interaction difficult. Naringenin is not deprotonated at pH 3.00, while CNA are poorly dispersed due to full protonation [36].

The structural changes of CNT can be estimated by Raman spectroscopy, which quantifies the density of structural defects and the effectivity of the functionalization process [37]. The Raman spectra of all samples showed the typical signals corresponding to the D (related to disorders and distortions of the carbon network) and G bands (graphitic bands related to the tangential mode associated with the sp^2^ hybridization carbons of the side graphitic walls of the CNT) [38]. The ratio between the intensity of the D and G bands (I_D_/I_G_) is a quantitative measurement of the density of defects and disorders in the lateral wall of the CNT. Consequently, the analysis of the I_D_/I_G_ ratio can be used to obtain information on structural changes that occur as the result of functionalization strategies, such as those involving chemical interactions [37]. The decrease in the I_D_/I_G_ ratio after the functionalization process is most likely due to the non-covalent interaction between Ngn and CNA. Small changes in the D band and the I_D_/I_G_ ratio were detected by the field disturbance and physical tension in the graphite skeleton, suggesting non-covalent interactions [39]. In addition, changes in the electronic environment caused by doping and chemical interactions influence the CNT’s Raman spectrum, specifically, the G band. This band represents pure graphite and shows high sensitivity to electronic effects [40]. The frequency of the G band peaks from CNANgn and CNANgn(ox) were higher than those from CNA and PM. The non-covalent interactions between molecules and the graphite skeleton increase the required energy for the occurrence of vibration. This event is reflected in the displacement of the G band to higher frequencies in the Raman spectrum [39]. Thus, this displacement and the decrease in the I_D_/I_G_ ratio for CNANgn and CNANgn(ox) suggested the non-covalent interaction between Ngn and the CNA, presumably hydrophobic interactions and hydrogen bonds. These interactions, specifically for CNANgn, were favored during the functionalization process by the lower availability of H^+^ at neutral pH.

In DSC curves, Ngn exhibited an endothermic melting peak indicating the crystalline nature of the compound. However, an imperceptible melting peak was found at the same temperature in CNANgn and CNANgn(ox) curves, indicating the Ngn changed from a crystalline to an amorphous structure. These changes can be attributed mainly to the deposition of the amorphous drug on the surface of the CNA during the functionalization process. The amorphous form had a lower enthalpy of fusion (Table 1). These results corroborate the findings from TEM and XPS. A PM curve presented the endothermic peak corresponding to the Ngn melting point, showing the presence of free Ngn. This peak appeared at a lower temperature, most likely due to the weak interaction between Ngn and CNA.

There are four types of adsorption sites available in and on the CNT: internal channels, an external surface, groove area, and interstitial pores [41,42]. However, only the outer surface of the CNT is generally available for adsorption. Based on the molecular structure of Ngn, with its aromatic rings and hydroxyl groups, we concluded that the molecules of Ngn were adsorbed non-covalently on the surface of the CNA through hydrogen bonds, weak electrostatic interactions (the most acidic proton on Ngn has a pKa value of ~7.1) [43], and π–π stacking.

The release test of Ngn from CNANgn and CNANgn(ox) at pH 7.4 and pH 5.5 simulates the biological fluids pH and the tumor periphery pH, respectively. The rate release of Ngn from the nanocarrier was pH-dependent with a higher at pH 7.4 than at pH 5.5. This could be attributed to the increased solubility of Ngn at the higher solution pH. At pH 7.4, Ngn would exist ~67% in its ionized, deprotonated state compared to only 2.5% at a solution pH of 5.5. Thus, solution pH plays an important role in the study of the Ngn release process.

The model with the highest coefficient of determination (r^2^) was the most appropriate kinetics model to describe the release of Ngn from CNT. The Ngn release mechanism was determined by analyzing the r^2^ value from kinetic models. Both samples, regardless of the pH conditions, presented the best fit with the Korsmeyer–Peppas model (r^2^ = 1) [44]. In the Korsmeyer–Peppas model, the release exponent (n) is indicative of the drug’s release mechanism. According to Korsmeyer’s theory, if n is 0.45, the drug release follows the Fickian diffusion mechanism. When 0.45 < *n* < 0.89, anomalous (non-Fickian) diffusion follows. However, for *n* = 0.89, the diffusion mechanism is the case II transport, and for n > 0.89 it is the super case II transport [45,46,47,48]. For our system with an n-value of 1, this points to the release being characterized by the transport of super case II. Therefore, the Ngn release rate can occur due to the diffusion and relaxation of the linked Ngn on the surface of the CNA [49,50].

The intracellular pH of tumor cells is slightly more alkaline than extracellular fluid, due to the exportation of lactic acid and protons by acid-base regulators [51,52]. The pH-responsive release behavior of CNANgn could aid the treatment of the tumor by prolonged-release properties, facilitating the delivery of higher concentrations of Ngn intracellularly and lower at the tumor’s periphery.

Concerning the cytocompatibility (hFB cells), free Ngn showed the highest cytotoxicity. CNANgn was less cytotoxic than Ngn, indicating that the functionalization promoted the decrease in drug cytotoxicity, leading to the possibility of reduced side effects. Both CNANgn and Ngn significantly inhibited the viability of A549 cells (malignant lung cells). The CC_50_ results indicate that CNANgn exhibits a higher cytotoxic activity than Ngn, meaning that Ngn has enhanced anticancer effects when functionalized to CNA. It is also important remember that the nanocomposite has lower than 50% of naringenin, since the nanocomposite formulation was obtained using 1:1 (NTCA:Ngn/w:w).

In conclusion, the nanocarrier seems to work synergistically as Ngn’s activity on malignant cells was observed at lower concentrations and reduced the drug toxicity on non-malignant cells when complexed with CNT. This sustained-release nanosystem could be used to increase the efficacy of pulmonary adenocarcinoma therapy with reduced side effects.

## 4. Materials and Methods

### 4.1. Materials

If not otherwise stated, all materials have been purchased from Sigma-Aldrich, (St. Louis, MO, USA). Double-distilled water was used after filtration in a Millipore system (home supplied).

### 4.2. Functionalization of CNT

Initial oxidation of CNT was performed via acid treatment, adapted from Cirillo et al. [38]. Briefly, CNT 1.5% (*w*/*v*) (MWCNTs, Sigma-Aldrich, St. Louis, MO, USA) was added to a 1:1 mixture of 15% nitric acid (*v*/*v*) (Labsynth, Diadema, São Paulo, Brazil) and 15% sulfuric acid (*v*/*v*) (Labsynth, Diadema, São Paulo, Brazil). The mixture was magnetically stirred (Mag-Multi, Marte, São Paulo, Brazil) for 24 h after the material was subjected to successive washes with distilled water until the solution maintained a pH of 7.0. The mixture was subsequently oven-dried at 100 °C for 24 h. (Mag-Multi, Marte, São Paulo, Brazil). This sample was called CNA. The functionalization reaction was carried out in the absence and presence of oxidative conditions. The first condition in absence of oxidative materials, CNANgn (10 mg/mL) and Ngn (10 mg/mL), the functionalized CNT and Ngn were stirred for 3 h at 25 °C in an aqueous solution. The preparation of the oxidative condition, CNANgn(ox), was similar to the first, however 1.8 mg/mL of ascorbic acid (Neon Comercial, Suzano, São Paulo Brazil) and hydrogen peroxide 7 mM (0.2 µL/mL, Labsynth, Diadema, São Paulo, Brazil) was added. A drug-free sample was also produced using the oxidative environment (CNA(ox)). Finally, the functionalized CNTs were dialyzed against distilled water (25 °C for 48 h) and dried in a TecnalTE-393/2 oven (Piracicaba, São Paulo, Brazil) (80 °C for 24 h). The physical mixture (PM) was prepared mixing the powders of CNA and Ngn in the same proportion (1:1/w:w).

### 4.3. Transmission Electron Microscopy (TEM)

TEM images were obtained using the JEOL 1011 TEM (Tokyo, Japan), operating at 60 kV acceleration. The images were imported into ImageJ Software version 1.8.0, and analyzed using an inhouse macro to obtain the diameter of the CNT (*n* = 15).

### 4.4. Infrared Spectroscopy with Fourier Transform (FTIR)

FTIR studies were carried out at room temperature in an FTIR-Bomen spectrophotometer (IRPrestige-21, Shimadzu, Kyoto, Japan) in the 400–4000 cm^−1^ range, using KBr pellets [53].

### 4.5. X-ray Photoelectron Spectroscopy (XPS)

X-ray photoelectron spectroscopy was performed with the Thermo K-Alpha XPS (Thermo Fisher Scientific, Waltham, MA, USA). The monochromatic X-ray source had an Al anode with Kα energy = 1486.6 eV [54,55].

### 4.6. Raman Spectroscopy

Raman spectra were recorded using a Confocal Raman spectrometer (Horiba Scientific, Model T64000, Horiba Scientific, Piscataway, NJ, USA) with excitation wavelength at 532 nm. Samples were prepared by deposition on a glass foil, and the spectra were recorded on a 20× objective [56].

### 4.7. Differential Scanning Calorimetry (DSC)

DSC was performed using the Shimadzu DSC-50 (Kyoto, Japan) equipment with a heating rate of 10 °C/min under nitrogen atmosphere. The temperature variation occurred between 30 °C and 500 °C and ~1.5 mg per sample was used in the analysis [57].

### 4.8. In Vitro Release Assay

The in vitro release of Ngn from the CNANgn and CNANgn(ox) was performed using the dialysis membrane diffusion method [30,58]. The samples were dissolved in phosphate buffer saline (PBS) at pH 7.4 and 5.5. Samples (1 mg/mL) were dissolved in PBS (0.01 M) and placed into the dialysis membranes (10 mL). The dialysis process was performed against 30 mL of PBS (receptor solution-stirring/37 °C). At predetermined time intervals, aliquots of 2 mL of the receptor solution were collected and read on the UV spectrophotometer (Biochrom Libra S22, Cambridge, UK, λ = 288 nm). The Ngn concentration was calculated against an appropriate Ngn calibration curve. To determine the kinetics of Ngn release from the CNA, the Korsmeyer–Peppas, Hixon–Crowell, Higuchi, and first-order kinetic models were used.

### 4.9. Cell Viability Assays

The effect of Ngn and CNANgn on the cells viability was evaluated using the MTT assay [59] in two distinct cell lines (hFB and A549), obtained from ATCC (PensabioBiotecnologia, São Paulo, Brazil). Consumables for cell culture were obtained from Sigma Chemical Co. (St. Louis, MO, USA). The cells (5 × 10^4^/well) were allowed to adhere to a 96-well tissue culture plates for 24 h at 37 °C in a 5% CO_2_ atmosphere. Non-adherent cells were removed by washes with sterile Dulbecco’s Modified Eagle Medium (DMEM) and the wells refilled with DMEM medium supplemented with 10% Fetal Bovine Serum (FBS). The cells were then incubated with increasing concentrations of the test compound (15.6 to 1000 µg) and incubated for an additional 48 h at 37 °C in a 5% CO_2_ atmosphere. The culture medium was discharged, and the formation of formazan was measured by addition of MTT (5 mg/mL in PBS, 25 µg/well), which was incubated for 3 h in the dark at 37 °C. The plates were then centrifuged at 500× *g* for 8 min, and the supernatant was removed. The pellet was dissolved in 200 µl of DMSO, and subsequently the absorbance was measured using a plate reader at 570 nm (SpectraMax Gemini 190, Molecular Devices, CA, USA). The 50% cytotoxicity inhibitory concentration (CC_50_) was determined by linear regression analysis using the Prism Graphpad 8 software.

### 4.10. Statistical Analysis

All assays were repeated three times to ensure reproducibility. The student’s t-test analyzed the statistical significance of the results obtained from the control and treated groups, using the Prism Graphpad 8 software. Differences are considered significant at a level of *p* < 0.05.

## 5. Conclusions

The present study has shown that the chemical functionalization of the CNT surface using the flavanone naringenin (Ngn) was done via non-covalent interactions. The neutral medium promoted the surface-functionalization of CNTs with Ngn through intermolecular interactions, predominantly through hydrogen bonds. In vitro release behavior of Ngn may contribute to improve the treatment of cancer with controlled and sustained release properties, which are pH-dependent. Finally, cytotoxicity was dependent on the concentration of the active agent and the cell type. CNANgn exhibited greater cytotoxicity against human lung alveolar adenocarcinoma (A549) cells compared to free drug, demonstrating the potential of CNANgn to inhibit the growth of lung cancer cells. At the same time, using CNANgn instead of free Ngn reduces the side effects, as there was a lower cytotoxicity of the healthy cell line (hFB) when using the CNANgn. This synergistic approach of the nanosystem can lead to increased efficacy of anticancer treatment of pulmonary adenocarcinomas, and should be validated in vivo. Despite CNA presenting cytotoxicity, after functionalization, this drug carrier was able to improve Ngn performance on tumor cells and decrease the toxicity of this drug and CNA on non-malignant cells.

## Figures and Tables

**Table 1:** Thermal parameters obtained from the DSC curves.

Sample	DSC Parameters		
	Initial Melting Temperature (°C)	Final Melting Temperature (°C)	Melting Enthalpy (J/g)
Ngn	251.7	255.2	−40.1
CNANng	194.8	262.7	−102.6
CNANng (ox)	204.7	259.3	−119.3
PM	226.2	255.4	−32.9

Ngn (Naringenin), CNANgn (CNA functionalized with naringenin), CNANgn(ox) (CNA functionalized with naringenin in oxidative environment).

**Table 2:** R^2^ values and kinetic parameters for mathematical modeling of Ngn release.

	R^2^ Value (r^2^)			
	CNANgn		CNANgn(ox)	
Mathematical Models	pH 5.5	pH 7.4	pH 5.5	pH 7.4
Korsmeyer–Peppas	1.000000	1.000000	1.000000	1.000000
Hixon–Crowell	0.999998	0.999998	0.999997	0.999998
Higuchi	0.892617	0.905022	0.885681	0.914770
First Order	0.999996	0.999996	0.999994	0.999996

## References

[1] Kanitkar A.A., Schwartz A.G., George J., Soubani A.O. (2018). Causes of death in long-term survivors of non-small cell lung cancer: A regional surveillance, epidemiology, and end results study. J. Ann. Thorac. Med..

[2] Zhou J., Sun M., Jin S., Fan L., Zhu W., Sui X., Cao L., Yang C., Han C. (2019). Combined using of paclitaxel and salinomycin active targeting nanostructured lipid carriers against non-small cell lung cancer and cancer stem cells. J. Drug Deliv..

[3] Cirillo G., Vittorio O., Kunhardt D., Valli E., Farfalla A., Curcio M., Spizzirri U.G., Hampel S. (2019). Combining carbon nanotubes and chitosan for the vectorization of methotrexate to lung cancer cells. J. Mater..

[4] Osmani L., Askin F., Gabrielson E., Li Q.K. (2018). Current WHO guidelines and the critical role of immunohistochemical markers in the subclassification of non-small cell lung carcinoma (NSCLC): Moving from targeted therapy to immunotherapy. In Proceedings of Seminars in Cancer Biology.

[5] Weeks J.C., Catalano P.J., Cronin A., Finkelman M.D., Mack J.W., Keating N.L., Schrag D. (2012). Patients’ expectations about effects of chemotherapy for advanced cancer. J. N. Engl. J. Med..

[6] Liu X., Xu D., Liao C., Fang Y., Guo B. (2018). Development of a promising drug delivery for formononetin: Cyclodextrin-modified single-walled carbon nanotubes. J. Drug Deliv. Sci. Technol..

[7] Hosnedlova B., Kepinska M., Fernandez C., Peng Q., Ruttkay-Nedecky B., Milnerowicz H., Kizek R. (2019). Carbon nanomaterials for targeted cancer therapy drugs: A critical review. J. Chem. Rec..

[8] Peng Z., Liu X., Zhang W., Zeng Z., Liu Z., Zhang C., Liu Y., Shao B., Liang Q., Tang W. (2020). Advances in the application, toxicity and degradation of carbon nanomaterials in environment: A review. J. Environ. Int..

[9] Chen Z., Zhang A., Wang X., Zhu J., Fan Y., Yu H., Yang Z. (2017). The advances of carbon nanotubes in cancer diagnostics and therapeutics. J. Nanomater..

[10] Saliev T. (2019). The advances in biomedical applications of carbon nanotubes. J. Carbon Res..

[11] Singh R.P., Sharma G., Singh S., Patne S.C., Pandey B.L., Koch B., Muthu M.S. (2016). Effects of transferrin conjugated multi-walled carbon nanotubes in lung cancer delivery. J. Mater. Sci. Eng. C.

[12] Costa P.M., Bourgognon M., Wang J.T., Al-Jamal K.T. (2016). Functionalised carbon nanotubes: from intracellular uptake and cell-related toxicity to systemic brain delivery. J. Control. Release.

[13] Liu Z., Robinson J.T., Tabakman S.M., Yang K., Dai H. (2011). Carbon materials for drug delivery & cancer therapy. J. Mater. Today.

[14] Patel K., Singh G.K., Patel D.K. (2018). A review on pharmacological and analytical aspects of naringenin. J. Chin. J. Integr. Med..

[15] Cavia-Saiz M., Busto M.D., Pilar-Izquierdo M.C., Ortega N., Perez-Mateos M., Muñiz P. (2010). Antioxidant properties, radical scavenging activity and biomolecule protection capacity of flavonoid naringenin and its glycoside naringin: A comparative study. J. Sci. Food Agric..

[16] Zaim Ö., Doğanlar O., Zreigh M.M., Doğanlar Z.B., Özcan H. (2018). Synthesis, Cancer-selective antiproliferative and apoptotic effects of some (±)-naringenin cycloaminoethyl derivatives. J. Chem Biodivers..

[17] Abotaleb M., Samuel S.M., Varghese E., Varghese S., Kubatka P., Liskova A., Büsselberg D. (2019). Flavonoids in cancer and apoptosis. J. Cancers.

[18] Chang H.L., Chang Y.M., Lai S.C., Chen K.M., Wang K.C., Chiu T.T., Chang F.H., Hsu L.S. (2017). Naringenin inhibits migration of lung cancer cells via the inhibition of matrix metalloproteinases-2 and-9. J. Exp. Ther. Med..

[19] Zhang H., Zhong X., Zhang X., Shang D., Zhou Y., Zhang C. (2016). Enhanced anticancer effect of ABT-737 in combination with naringenin on gastric cancer cells. J. Exp. Ther. Med..

[20] Unsalan O., Erdogdu Y., Gulluoglu M.T. (2009). FT-Raman and FT-IR spectral and quantum chemical studies on some flavonoid derivatives: Baicalein and naringenin. J. Raman Spectrosc..

[21] Cirillo G., Vittorio O., Hampel S., Iemma F., Parchi P., Cecchini M., Puoci F., Picci N. (2013). Quercetin nanocomposite as novel anticancer therapeutic: improved efficiency and reduced toxicity. Eur. J. Pharm. Sci..

[22] Nia A.H., Rezaeian S., Eshghi H., Haghbeen K., Bakavoli M., Ramezani M. (2018). Synthesis and evaluation of apoptosis induction levels of carbamate-and thiocarbamate-functionalized multi-walled carbon nanotubes. J. Iran. Chem. Soc..

[23] Gómez S., Rendtorff N.M., Aglietti E.F., Sakka Y., Suárez G. (2016). Surface modification of multiwall carbon nanotubes by sulfonitric treatment. J. Appl. Surf. Sci..

[24] Yudianti R., Onggo H., Saito Y., Iwata T., Azuma J.-i. (2011). Analysis of functional group sited on multi-wall carbon nanotube surface. Open Mater. Sci. J..

[25] Bilalis P., Katsigiannopoulos D., Avgeropoulos A., Sakellariou G. (2014). Non-covalent functionalization of carbon nanotubes with polymers. RSC Adv..

[26] Zawawi N.A., Majid Z.A., Aini N., Rashid A. (2016). Effect of acid oxidation methods on multi-walled carbon nanotubes (MWCNT) for drug delivery application. Int. J. Adv. Sci. Res. Manag..

[27] Osorio A., Silveira I., Bueno V., Bergmann C. (2008). H_2_SO_4_/HNO_3_/HCl—Functionalization and its effect on dispersion of carbon nanotubes in aqueous media. J. Appl. Surf. Sci..

[28] Maity S., Mukhopadhyay P., Kundu P.P., Chakraborti A.S. (2017). Alginate coated chitosan core-shell nanoparticles for efficient oral delivery of naringenin in diabetic animals-An in vitro and in vivo approach. Carbohydr. Polym..

[29] Ji P., Yu T., Liu Y., Jiang J., Xu J., Zhao Y., Hao Y., Qiu Y., Zhao W., Wu C. (2016). Naringenin-loaded solid lipid nanoparticles: preparation, controlled delivery, cellular uptake, and pulmonary pharmacokinetics. Drug Des. Dev. Ther..

[30] Razzazan A., Atyabi F., Kazemi B., Dinarvand R. (2016). In vivo drug delivery of gemcitabine with PEGylated single-walled carbon nanotubes. Mater. Sci. Eng. C.

[31] Gera S., Talluri S., Rangaraj N., Sampathi S. (2017). Formulation and evaluation of naringenin nanosuspensions for bioavailability enhancement. AAPS PharmSciTech.

[32] Kumar S.P., Birundha K., Kaveri K., Devi K.T. (2015). Antioxidant studies of chitosan nanoparticles containing naringenin and their cytotoxicity effects in lung cancer cells. Int. J. Biol. Macromol..

[33] Wang Y., Ma J., Zhu J., Ye N., Zhang X., Huang H. (2016). Multi-walled carbon nanotubes with selected properties for dynamic filtration of pharmaceuticals and personal care products. Water Res..

[34] Scheibe B., Borowiak-Palen E., Kalenczuk R.J. (2010). Oxidation and reduction of multiwalled carbon nanotubes —preparation and characterization. Mater. Charact..

[35] Wang N., Feng Y., Zeng L., Zhao Z., Chen T. (2015). Functionalized multiwalled carbon nanotubes as carriers of ruthenium complexes to antagonize cancer multidrug resistance and radioresistance. ACS Appl. Mater. Interfaces.

[36] Zhu H., Poojary M.M., Andersen M.L., Lund M.N. (2019). Effect of pH on the reaction between naringenin and methylglyoxal: A kinetic study. Food Chem..

[37] Wepasnick K.A., Smith B.A., Bitter J.L., Howard Fairbrother D. (2010). Chemical and structural characterization of carbon nanotube surfaces. Anal. Bioanal. Chem..

[38] Cirillo G., Hampel S., Klingeler R., Puoci F., Iemma F., Curcio M., Parisi O.I., Spizzirri U.G., Picci N., Leonhardt A. (2011). Antioxidant multi-walled carbon nanotubes by free radical grafting of gallic acid: New materials for biomedical applications. J. Pharm. Pharmacol..

[39] Yoo S., Hou J., Yi W., Li Y., Chen W., Meng L., Si J., Hou X. (2017). Enhanced response of metformin towards the cancer cells due to synergism with multi-walled carbon nanotubes in photothermal therapy. Sci. Rep..

[40] Rathod V., Tripathi R., Joshi P., Jha P.K., Bahadur P., Tiwari S. (2019). Paclitaxel encapsulation into dual-functionalized multi-walled carbon nanotubes. AAPS PharmSciTech.

[41] Mehra N.K., Palakurthi S. (2016). Interactions between carbon nanotubes and bioactives: A drug delivery perspective. Drug Discov. Today.

[42] Foldvari M., Bagonluri M. (2008). Carbon nanotubes as functional excipients for nanomedicines: II. drug delivery and biocompatibility issues. Nanomed. Nanotechnol. Biol. Med..

[43] Zhang L., Song L., Zhang P., Liu T., Zhou L., Yang G., Lin R., Zhang J. (2015). Solubilities of naringin and naringenin in different solvents and dissociation constants of naringenin. J. Chem. Eng. Data.

[44] Costantini L., Bouropoulos N., Fatouros D.G., Kontopoulou I., Roldo M. (2016). Synthesis of carbon nanotubes loaded hydroxyapatite: Potential for controlled drug release from bone implants. J. Adv. Ceram..

[45] Vieira R., Severino P., Nalone L.A., Souto S.B., Silva A.M., Lucarini M., Durazzo A., Santini A., Souto E.B. (2020). Sucupira oil-loaded nanostructured lipid carriers (NLC): Lipid screening, factorial design, release profile, and cytotoxicity. Molecules.

[46] Souto E.B., Zielinska A., Souto S.B., Durazzo A., Lucarini M., Santini A., Silva A.M., Atanasov A.G., Marques C., Andrade L.N. (2020). (+)-Limonene 1,2-epoxide-loaded SLN: evaluation of drug release, antioxidant activity and cytotoxicity in HaCaT cell line. Int. J. Mol. Sci..

[47] Souto E.B., Souto S.B., Zielinska A., Durazzo A., Lucarini M., Santini A., Horbańczuk O.K., Atanasov A.G., Marques C., Andrade L.N. (2020). Perillaldehyde 1,2-epoxide loaded SLN-tailored mAb: Production, physicochemical characterization and in vitro cytotoxicity profile in MCF-7 cell lines. Pharmaceutics.

[48] Ferreira Marques C.S., Rezende P., Andrade L.N., Mendes T.M.F., Allegretti S.M., Bani C., Chaud M.V., Batista de Almeida M., Souto E.B., da Costa L.P. (2018). Solid dispersion of praziquantel enhanced solubility and improve the efficacy of the schistosomiasis treatment. J. Drug Deliv. Sci. Technol..

[49] Peppas N.A., Sahlin J.J. (1989). A simple equation for the description of solute release. III. Coupling of diffusion and relaxation. Int. J. Pharm..

[50] Mahajan C.R., Joshi L.B., Varma U., Naik J.B., Chaudhari V.R., Mishra S. (2019). Sustainable drug delivery of famotidine using chitosan-functionalized graphene oxide as nanocarrier. Glob. Chall. (Hoboken, NJ).

[51] Webb B.A., Chimenti M., Jacobson M.P., Barber D.L. (2011). Dysregulated pH: A perfect storm for cancer progression. Nat. Rev. Cancer.

[52] Griffiths J.R., McIntyre D.J., Howe F.A., Stubbs M. (2001). Why are cancers acidic? A carrier-mediated diffusion model for H+ transport in the interstitial fluid. Novartis Found. Symp..

[53] Ling X., Wei Y., Zou L., Xu S. (2013). The effect of different order of purification treatments on the purity of multiwalled carbon nanotubes. J. Appl. Surf. Sci..

[54] Sezer N., Koc M. (2019). Oxidative acid treatment of carbon nanotubes. J. Surf. Interfaces.

[55] Luo X., Matranga C., Tan S., Alba N., Cui X.T. (2011). Carbon nanotube nanoreservior for controlled release of anti-inflammatory dexamethasone. J. Biomater..

[56] Clancy A.J., White E.R., Tay H.H., Yau H.C., Shaffer M.S. (2016). Systematic comparison of conventional and reductive single-walled carbon nanotube purifications. J. Carbon.

[57] Da Silva L.R., Martins L.d.V., Calou I.B.F., de Deus M.d.S.M., Ferreira P.M.P., Peron A.P. (2015). Flavonóides: constituição química, ações medicinais e potencial tóxico. J. Acta Toxicológica Argent..

[58] Zhao X., Tian K., Zhou T., Jia X., Li J., Liu P. (2018). PEGylated multi-walled carbon nanotubes as versatile vector for tumor-specific intracellular triggered release with enhanced anti-cancer efficiency: optimization of length and PEGylation degree. Colloids Surf. B Biointerfaces.

[59] Rigon R.B., Goncalez M.L., Severino P., Alves D.A., Santana M.H.A., Souto E.B., Chorilli M. (2018). Solid lipid nanoparticles optimized by 2(2) factorial design for skin administration: Cytotoxicity in NIH3T3 fibroblasts. Colloids Surf. B Biointerfaces.

